# Solitary living as a risk factor for maxillofacial space infections: Insights from a case-control study

**DOI:** 10.1097/MD.0000000000042978

**Published:** 2025-06-27

**Authors:** Jilun Liu, Shuning Li, Wei Yang, Xuhui Fan

**Affiliations:** a The Second Hospital of Hebei Medical University, Shijiazhuang, Hebei Province, China.

**Keywords:** educational level, maxillofacial space infections, risk factors, solitary living

## Abstract

Odontogenic infections are a major contributor to the occurrence of maxillofacial space infections (MSI) worldwide. Despite effective management, MSI can lead to severe complications. This study aimed to identify patient characteristics, treatment strategies, and outcomes associated with MSI in a specialized healthcare setting, focusing on risk factors for life-threatening complications. In this case-control study, we analyzed 116 medical records and surveyed 116 healthy individuals. Data collected included gender, age, body mass index (BMI), presence of diabetes, hypertension, coronary artery disease, cohabitation status with children or parents, and educational level. For patients with MSI, clinical symptoms, treatment courses, and hospitalization duration were documented. Patients with MSI were predominantly aged 50 to 70 and had higher BMIs (*P* < .05), with 57.76% being male. Only 33.62% of these patients cohabited with children or parents (*P* < .01), and many had lower educational levels (*P* < .01). Higher BMI, solitary living (not cohabiting with children or parents), and lower educational levels independently increased the risk of MSI. The influence of the home environment on MSI warrants further attention.

## 1. Introduction

Cellulitis is a condition that affects the deep layers of the skin, including the dermis and subcutaneous tissues, typically occurring when harmful microorganisms penetrate the dermis through skin openings or wounds. The lack of timely and effective treatment, impairment of host resistance, and the unique anatomy of the maxillofacial region can cause localized odontogenic infections to rapidly progress into maxillofacial space infections (MSI). Severe MSI are often associated with complications such as sepsis, respiratory depression, necrotizing fasciitis, and chest infections, resulting in prolonged and costly treatment.^[1]^ This poses a significant threat to public health due to its potential fatality. Although localized odontogenic infection is a major contributing factor to MSI, other factors such as family relationships and educational level may also play a role, yet these remain underexplored.^[2]^

The objective of this study was to analyze the clinical features of MSI, assess their potential association with family relationships and educational level, and identify factors that affect the duration of hospitalization following abscess incision. Additionally, this study is the first to report on the impact of solitary living on MSI.

## 2. Methods

### 2.1. Study design and sample

This retrospective case-control study was conducted on all patients with MSI treated at the Department of Oral and Maxillofacial Surgery from June 1, 2023, to May 31, 2024. The study focused exclusively on odontogenic infections that had spread to the soft tissues of the maxillofacial region, excluding non-odontogenic infections and those confined to hard tissues. Complete anonymity of the data was maintained, and an application for a waiver of informed consent was approved by the ethics committee. The data access period was from August 25, 2024, to August 31, 2024. Patients with MSI were subject to uniform treatment protocols and admission criteria, which included swelling affecting vital anatomical structures, persistent fever, disease progression despite anti-inflammatory treatment, and the necessity for abscess incision and drainage.

### 2.2. Setting and participants

The study was conducted in the Department of Oral and Maxillofacial Surgery. A comprehensive review of medical records was undertaken for individuals diagnosed with odontogenic MSI and treated in this department. A total of 116 patient cases were included. To identify fundamental infectious factors, 116 healthy controls were randomly selected from various city locations. Eligibility criteria for cases and controls included factors such as age, gender, BMI, presence of diabetes, hypertension, coronary heart disease, cohabitation status with children or parents, and educational level (ranging from illiteracy to graduate and above). For MSI patients, additional data on clinical symptoms, treatment courses, and hospitalization length were collected. Previous studies have shown the importance of understanding the epidemiology and management of odontogenic infections.^[3]^ Additionally, the role of systemic health factors in oral infections has been explored in recent literature.^[4]^

### 2.3. Data collection, management, and analysis

Data were documented using standardized collection forms. A database was created with Microsoft Excel and imported into R for statistical analysis. Descriptive statistics were computed for all variables. Univariate analyses were conducted to explore correlations between factors and life-threatening complications, with odds ratios and *P*-values calculated, considering a *P*-value <.05 as statistically significant. Multivariate logistic regression was applied to further analyze significant risk factors.

Statistical methods included controlling for confounding variables through multivariate analyses, addressing potential bias by randomizing control selection, and employing sensitivity analyses to validate findings. Missing data were managed through case-wise deletion. The study size was determined by the total number of eligible patients treated within the specified period, ensuring adequate power to detect significant associations.

## 3. Results

This study contained a cohort of 232 individuals who had comprehensive medical records. The basic features of these individuals are shown in Table 1.

**Table 1:** Fundamental data on the individuals included in this research.

	Frequency	Percentage
Gender		
Male	128	55.17
Female	104	44.83
Age		
Age < 15	19	8.19
15≤age < 25	16	6.90
25≤age < 35	19	8.19
35≤age < 45	31	13.36
45≤age < 55	37	15.95
55≤age < 65	42	18.10
65≤age < 75	52	22.41
75≤age < 85	14	6.03
Age≥85	2	0.86
Diabetes		
Yes	53	22.84
No	179	77.16
Hypertension		
Yes	68	29.31
No	164	70.69
Coronary heart disease		
Yes	22	9.48
No	210	90.52
Cohabitation with children (or parents)		
Yes	124	53.45
No	108	46.55
Educational level		
Illiteracy	44	18.97
Elementary school	63	27.16
Junior high school	46	19.83
High school	35	15.09
Undergraduate	37	15.95
Graduate and above	7	3.02

### 3.1. General condition of the patient

**Figure 1.**
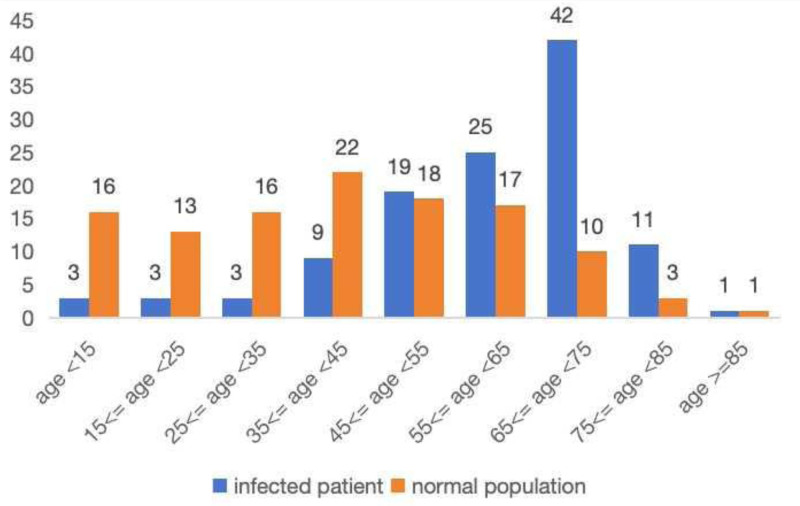
Distribution of patients according to age.

In this study, 67 (57.76%) of the infected patients were men, whereas 61 (52.59%) of the normal population were males. The *P*-value for the comparison between the 2 groups is .428. The body mass index (BMI) was 25.52 (24.53–25.95) in patients with infections and 22.02 (21.58–22.45) in the general population. The difference in BMI between the 2 groups was statistically significant, with a *P*-value of <.001. This indicates that obesity might be a contributing factor in the development of MSI. The mean age of infected patients was 61.5 years (range: 51–70.25), whereas the mean age of the normal population was 39 years (range: 24.5–56.25). The difference in age between the 2 groups was statistically significant, with a *P*-value of <.001. These findings indicate that advancing age may be a contributing factor in the development of MSI (Fig. 1).

### 3.2. Presence of concomitant diseases (diabetes, hypertension, coronary heart disease)

The number of diabetes mellitus, hypertension and coronary artery disease in infected patients were 38 (32.76%), 45 (38.79%), and 13 (11.21%), respectively, and in the normal population were 15 (12.93%), 23 (19.83%), 9 (7.76%). The *P*-value was <.05 in the patients suffering from diabetes mellitus and hypertension, which suggests that diabetes mellitus and hypertension may be one of the influencing factors in the development of MSI.

### 3.3. Cohabitation with children (or parents) and educational level

73.28% (85) of the normal population lived with their children or parents, which was significantly >33.62% (39) of the infected patients, *P* < .001.

**Figure 2.**
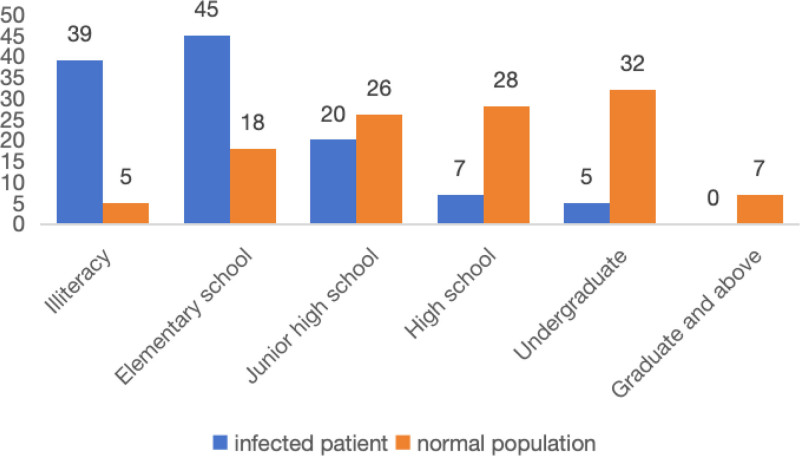
Patients categorized based on their degree of education.

The patients were classified into several categories based on their educational attainment, including illiteracy, elementary school, junior high school, high school, undergraduate, graduate, and postgraduate levels. The education level of the general population was much greater compared to that of the MSI patients, as seen in Figure 2.

### 3.4. Independent influencing factors

Following a univariate examination of the aforementioned various characteristics, it was ultimately concluded that BMI, age, presence of diabetes mellitus, presence of hypertension, living with children or parents, and educational level may potentially be the influencing factors for MSI, as shown in Table 2.

**Table 2:** Patient characteristics and presence of infection – univariate analysis.

	The normal population	Infected patient	Statistic	*P*-value
Age	39 (24.5–56.25)	61.5 (51–70.25)	−6.95	<.001
BMI	22.02 (21.58–22.45)	25.52 (24.53–25.95)	−9.93	<.001
Gender				
Male	61 (52.59%)	67 (57.76%)	0.63	.428
Female	55 (47.41%)	49 (42.24%)		
Diabetes				
Yes	15 (12.93%)	38 (32.76%)	12.94	<.001
No	101 (87.07%)	78 (67.24%)		
Hypertension				
Yes	23 (19.83%)	45 (38.79%)	10.07	.002
No	93 (80.17%)	71 (61.21%)		
Coronary heart disease				
Yes	9 (7.76%)	13 (11.21%)	0.80	.370
No	107 (92.24%)	103 (88.79%)		
Cohabitation with children (or parents)				
Yes	85 (73.28%)	39 (33.62%)	36.66	<.001
No	31 (26.72%)	77 (66.38%)		
Educational level				
Illiteracy	5 (4.31%)	39 (33.62%)	76.47	<.001
Elementary school	18 (15.52%)	45 (38.79%)		
Junior high school	26 (22.41%)	20 (17.24%)		
High school	28 (24.13%)	7 (6.03%)		
Undergraduate	32 (27.59%)	5 (4.31%)		
Graduate and above	7 (6.03%)	0		

When calculating differences in age and BMI, the method used was a 2-independent sample nonparametric test, and the statistic was the *Z*-value.

Differences in gender, diabetes, hypertension, coronary heart disease, and cohabitation with children or parents were calculated using the chi-square test, with a chi-square value as the statistical measure.

Differences in literacy level were calculated using the k-independent samples rank sum test with a chi-square statistic.

BMI = body mass index.

**Figure 3.**
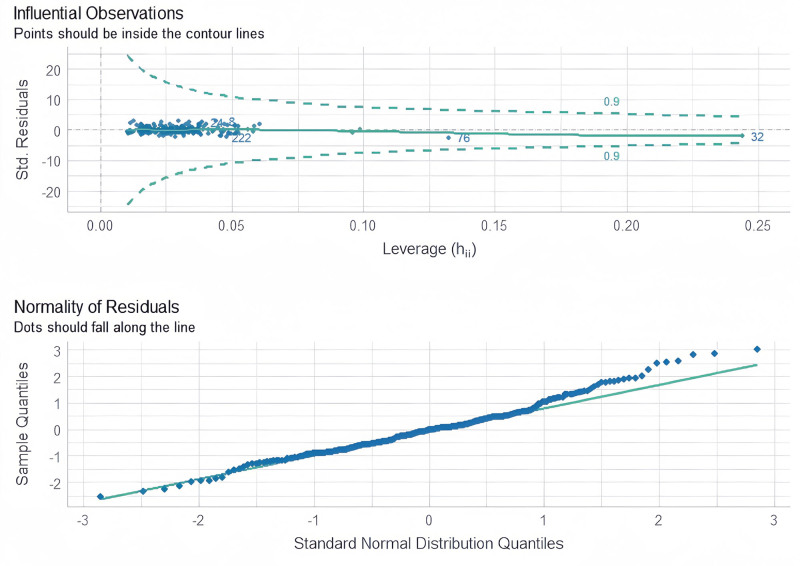
Patient characteristics and presence of infection – model test.

We conducted a more detailed multifactor analysis (Table 3) and tested the model (Fig. 3).

**Table 3:** Patient characteristics and presence of infection – multivariate analysis.

	Estimate	Std. error	*Z*-value	*Pr*...*z*	OR (95% CI)
(Intercept)	−14.9	2.793	−5.335	<.01	0 (0–0)
BMI	0.741	0.123	6.045	<.01	2.098 (1.679–2.724)
Age	0.014	0.011	1.211	.226	1.014 (0.992–1.037)
Diabetes					
Yes	0.766	0.587	1.304	.192	2.151 (0.682–6.929)
No	0				1
Hypertension					
Yes	0.242	0.56	0.432	.666	1.274 (0.422–3.856)
No	0				1
Cohabitation with children (or parents)					
Yes	−1.109	0.469	−2.363	.018	0.33 (0.129–0.824)
No	0				1
Educational level					
	−0.899	0.186	−4.823	<.01	0.407 (0.276–0.576)

CI = confidence interval, OR = odds ratio.

In the multifactorial analysis, only BMI OR 2.098 (95% CI: 1.679–2.724), cohabitation with children or parents OR 0.33 (95% CI: 0.129–0.824) and literacy level OR 0.407 (95% CI: 0.276–0.576) were validated as independent influences on the occurrence of MSI.

The correlation coefficient of BMI was >0. The larger the patient’s BMI was, the more likely to develop MSI; the correlation coefficient of child or parent cohabitation and literacy level was <0, the less likely to develop MSI when living with children or parents and having a high level of literacy; in which the risk of developing MSI was 33% higher when living with children or parents than when not living with children or parents.

### 3.5. Multifactorial analysis of length of stay

For the duration of hospitalization of infected patients, we also performed a multifactorial analysis in an attempt to find out the factors influencing the prolongation of hospitalization (Table 4).

**Table 4:** Patient characteristics and length of stay – multivariate analysis.

	Estimate	Std. error	*t*-value	*Pr*(> *t* )	vif
(Intercept)	−0.833	4.577	−0.182	.856	–
BMI	0.226	0.167	1.352	.18	1.066
Age	0.097	0.032	3.038	.003	1.091
Gender	−0.256	1.04	−0.246	.806	1.051
Diabetes	3.495	1.19	2.938	.004	1.209
Hypertension	0.219	1.223	0.179	.858	1.378
Coronary heart disease	1.878	1.775	1.058	.293	1.101
*F*_(9,82)_	3.706				
*P*	<.001				
Adjusted *R*-squared	0.211				
Implicit variable: length of stay					

vif = variance inflation factor.

Among them, age and diabetes were influential factors in prolonging hospitalization. More extended hospital stays with older age and diabetes.

## 4. Discussion

In our clinical work, we have found that patients with MSI tend to have a low level of education and do not live with other family members. Moreover, we have analyzed this situation in detail, which has not been mentioned in any other study. There was a 33% increase in the risk of developing MSI when not living with children or parents and a 40% decrease in the risk of developing MSI for each level of education. We believe that the low level of education of the patients and the fact that they lived alone, on the 1 hand, led to low awareness of oral health and the simple belief that toothache can be treated by taking painkillers or anti-inflammatory drugs. Even if left untreated, will not lead to severe consequences. And that this requires a concerted effort to strengthen social education, popularize knowledge of oral health, and improve the economic level of the people; on the other hand, it led to a low level of the economic status of the patients, and that the patients were at a higher risk of suffering from MSI. In rural elderly populations, the probability of low-income individuals suffering from 3 or more chronic diseases is 2.1 times that of their middle-income counterparts. This compounded effect results in annual medical expenditures exceeding 75% of their income, thereby establishing a vicious cycle of “disease and poverty.”^[5]^ Reluctance to undergo sound oral treatment when the cost of oral treatment is high leaves the infection unchecked in the early stages of its development.

Patients with MSI are predominantly male in terms of gender, and females may be more inclined towards oral care.^[6]^ Possible reasons: poor oral hygiene^[7]^ limited knowledge leading to poor oral health awareness.^[8]^ Patients tend to live alone.^[9,10]^ These factors together lead to the fact that patients often fail to pay enough attention to the disease in its early stages, and by the time they visit our clinic, it is already severe.

MSI occurs in what we consider to be an acute manifestation of a chronic condition, and often, patients have a long history of pain or swelling.^[11]^ Ewa Zawiślak observed that despite the recurrent nature of the condition, the vast majority of patients only become concerned when swelling suddenly develops and fails to improve on its own.^[12]^ When swelling occurs in superficial areas, patients recover quickly after abscess incision and drainage with aggressive surgical dressing changes; when swelling occurs in deep tissues (e.g., parapharyngeal space,^[13]^ pterygomandibular space^[14]^), it may be accompanied by respiratory depression, which triggers simultaneous infections in multiple spaces, which can be life-threatening.^[12,15–17]^

In severe cases, tracheotomy is required.^[18]^ In patients with airway obstruction, we prefer tracheotomy to tracheal intubation to maintain the airway. A study by Potter et al^[19]^ has shown that patients with deep neck infections who undergo tracheotomy for airway management spend less time in intensive care, have lower rates of complications, and incur lower costs when compared to patients who undergo endotracheal intubation. Patients who underwent tracheotomy for airway management in our study made an uneventful recovery.

There has been increasing concern about antibiotic-resistant bacteria, such as community-acquired methicillin-resistant S aureus (MRSA),^[20,21]^ which is reflected in the increased use of anti-MRSA antibiotics (e.g., vancomycin, trimethoprim-sulfamethoxazole, doxycycline, clindamycin) and broad-spectrum gram-negative antibiotics (e.g., β-lactam/β-lactamase inhibitors, levofloxacin, ceftriaxone) during the past decade.^[22,23]^ A bacterial culture of the pus from the abscess incision and targeted antibiotic administration is generally considered the optimal protocol. However, the cultures are negative in most cases.^[24]^ A tiny number of bacteria induces a powerful inflammatory response, or the immune system or antibiotic application reduces the number of viable bacteria to a deficient number.^[25–27]^ Bacterial toxins and other inflammatory mediators that trigger an escalating inflammatory response may better define the pathogenesis of cellulitis than the bacterial load itself. Empirical antibiotic therapy at this time can also yield good clinical results.^[28]^

In the present study, 32.76% of the patients had diabetes mellitus, and 38.79% of the patients had hypertension, which was significantly higher than the normal population (*P* < .05). Diabetes mellitus and hypertension are indeed influencing factors in MSI. Consistent with the findings of other studies.^[29–32]^ Immunologic studies have shown some defects in the host immune mechanism in diabetic patients.^[33]^ Polymorphonuclear leukocytes exhibit impaired migration, phagocytosis, intracellular killing and chemotaxis.^[34]^ In addition to widespread impaired immune function, certain nonimmune factors contribute to an increased risk of infection. Vascular abnormalities, such as microangiopathy and macrovascular disease,^[35]^ favor infection by disrupting local circulation, leading to death. However, diabetes mellitus and hypertension failed to manifest as independent influences on maxillofacial cellulitis in the multivariate, suggesting that good glycemic and blood pressure control can be achieved without catalyzing the development of MSI. However, once MSI occurs in a diabetic patient, the infection tends to spread rapidly, and even with reasonable diabetes control, there is still a high chance of severe infection.^[36,37]^

When patients are obese, they also have an increased prevalence of maxillofacial cellulitis, which is consistent with the studies of Garg et al,^[29]^ Zacay et al,^[32]^ and Rasteniene et al.^[30]^

Acknowledging the study’s limitations, the cross-sectional design precludes establishing causal relationships, necessitating future large-scale cohort studies for further verification. The influence of potential confounders, such as medication use and other comorbidities, cannot be fully excluded despite adjusting for some relevant variables.

## 5. Conclusions

In conclusion, maintaining a healthy body weight, living with family, and achieving a higher education level are effective ways to reduce MSI incidence from a socioeconomic perspective.

## Acknowledgments

The authors thank all the oral and maxillofacial surgeons and immunologists for their contribution to making the information easily accessible.

## Author contributions

**Conceptualization:** Jilun Liu.

**Data curation:** Shuning Li.

**Formal analysis:** Shuning Li, Wei Yang, Jilun Liu.

**Funding acquisition:** Jilun Liu.

**Methodology:** Shuning Li.

**Project administration:** Wei Yang.

**Software:** Xuhui Fan.

**Supervision:** Xuhui Fan.

**Validation:** Jilun Liu.

**Writing – original draft:** Shuning Li, Jilun Liu.

**Writing – review & editing:** Shuning Li, Jilun Liu.
